# Differential Pathogenesis of Lung Adenocarcinoma Subtypes Involving Sequence Mutations, Copy Number, Chromosomal Instability, and Methylation

**DOI:** 10.1371/journal.pone.0036530

**Published:** 2012-05-10

**Authors:** Matthew D. Wilkerson, Xiaoying Yin, Vonn Walter, Ni Zhao, Christopher R. Cabanski, Michele C. Hayward, C. Ryan Miller, Mark A. Socinski, Alden M. Parsons, Leigh B. Thorne, Benjamin E. Haithcock, Nirmal K. Veeramachaneni, William K. Funkhouser, Scott H. Randell, Philip S. Bernard, Charles M. Perou, D. Neil Hayes

**Affiliations:** 1 Lineberger Comprehensive Cancer Center, University of North Carolina at Chapel Hill, Chapel Hill, North Carolina, United States of America; 2 Department of Biostatistics, University of North Carolina at Chapel Hill, Chapel Hill, North Carolina, United States of America; 3 Department of Statistics and Operations Research, University of North Carolina at Chapel Hill, Chapel Hill, North Carolina, United States of America; 4 Department of Pathology and Laboratory Medicine, University of North Carolina at Chapel Hill, Chapel Hill, North Carolina, United States of America; 5 Department of Internal Medicine, Division of Medical Oncology, Multidisciplinary Thoracic Oncology Program, University of North Carolina at Chapel Hill, Chapel Hill, North Carolina, United States of America; 6 Department of Surgery, Division of Cardiothoracic Surgery, University of North Carolina at Chapel Hill, Chapel Hill, North Carolina, United States of America; 7 Department of Cell and Molecular Physiology, University of North Carolina at Chapel Hill, Chapel Hill, North Carolina, United States of America; 8 Department of Genetics, University of North Carolina at Chapel Hill, Chapel Hill, North Carolina, United States of America, University of North Carolina at Chapel Hill, University of North Carolina at Chapel Hill, Chapel Hill, North Carolina, United States of America, Chapel Hill, North Carolina, United States of America; 9 Utah Health Sciences Center, Salt Lake City, Utah, United States of America; Institut Gustave Roussy, France

## Abstract

**Background:**

Lung adenocarcinoma (LAD) has extreme genetic variation among patients, which is currently not well understood, limiting progress in therapy development and research. LAD intrinsic molecular subtypes are a validated stratification of naturally-occurring gene expression patterns and encompass different functional pathways and patient outcomes. Patients may have incurred different mutations and alterations that led to the different subtypes. We hypothesized that the LAD molecular subtypes co-occur with distinct mutations and alterations in patient tumors.

**Methodology/Principal Findings:**

The LAD molecular subtypes (Bronchioid, Magnoid, and Squamoid) were tested for association with gene mutations and DNA copy number alterations using statistical methods and published cohorts (*n* = 504). A novel validation (*n = *116) cohort was assayed and interrogated to confirm subtype-alteration associations. Gene mutation rates (*EGFR, KRAS, STK11, TP53*), chromosomal instability, regional copy number, and genomewide DNA methylation were significantly different among tumors of the molecular subtypes. Secondary analyses compared subtypes by integrated alterations and patient outcomes. Tumors having integrated alterations in the same gene associated with the subtypes, e.g. mutation, deletion and underexpression of *STK11* with Magnoid, and mutation, amplification, and overexpression of *EGFR* with Bronchioid. The subtypes also associated with tumors having concurrent mutant genes, such as *KRAS-STK11* with Magnoid. Patient overall survival, cisplatin plus vinorelbine therapy response and predicted gefitinib sensitivity were significantly different among the subtypes.

**Conclusions/ Significance:**

The lung adenocarcinoma intrinsic molecular subtypes co-occur with grossly distinct genomic alterations and with patient therapy response. These results advance the understanding of lung adenocarcinoma etiology and nominate patient subgroups for future evaluation of treatment response.

## Introduction

Lung cancer is the leading cause of cancer deaths worldwide [1] with lung adenocarcinoma (LAD) being one of the most common morphological varieties. Recently, reports of DNA copy number [2]–[5], gene sequence mutation [2], [3], [6]–[8], DNA methylation [9], and gene expression [2]–[4], [6]–[8], [10]–[18] have revealed LAD to be among the most heavily mutated and genomically-altered cancers [7]. However, few genes are commonly mutated, for example *TP53* is the most frequent at only 35% [6]. This lack of a universal mutation pattern indicates that LAD's molecular pathogenesis has abundant variety. Some of this variety, such as the mutual exclusivity of *EGFR* mutation and *KRAS* mutation has been established and has led to therapeutic advances [6], [19].

A more complete understanding of LAD's molecular pathogenesis is clinically-relevant because it could lead to better application and development of targeted therapies [19], [20]. Currently, targeted chemotherapy is administered to LAD patients having a particular genomic alteration, e.g. *EGFR* inhibitors (erlotinib, gefitinib) for *EGFR* mutant cancers [19], [21]. However, many studies have found that about 20% of patients with mutant *EGFR* fail to respond to *EGFR* inhibitors and about 25% of patients with wildtype *EGFR* receive clinical benefit from *EGFR* inhibitors [22], [23]. So, factors beyond one genomic alteration can affect response to targeted therapy. Identifying these additional factors could improve patient outcomes.

Using genome-wide gene expression profiling, LAD has been divided into intrinsic molecular subtypes by many investigators [3], [8], [10]–[16] including a meta-analysis by our group [14], [21] in which we named the molecular subtypes: Bronchioid, Magnoid, and Squamoid. The subtypes represent the main naturally-occurring patterns of LAD gene expression and separate tumors following different functional pathways, such as proliferation in Magnoid and development in Bronchioid. The subtypes are also clinically-relevant, as the Bronchioid subtype predicts superior patient survival [14]. Considering that different genomic alterations in LAD mouse models cause divergent gene expression [24], we hypothesized that tumors in different molecular subtypes would have distinct alterations in gene sequence, DNA copy number, and DNA methylation and combinations thereof. Beyond preliminary reports of *EGFR* and *KRAS* sequence mutations with subtype [2], [3], [16], LAD subtype-specific alterations are unknown.

Here, we detected genomic alterations co-occurring with the LAD molecular subtypes using published discovery cohorts [2], [6], [8], [11]. To independently validate these associations, we collected a novel cohort of patients with LAD (*n* = 116) and assayed their tumors for genome-wide gene expression, genome-wide DNA copy number, genome-wide DNA methylation, and selected gene sequence mutations. For the first time, we demonstrate that LAD molecular subtypes correlate with grossly distinct genomic alterations and patient therapy response.

## Materials and Methods

### Study design and published cohorts

A discovery and validation study design was followed to detect and validate genomic alterations associated with the LAD molecular subtypes. Published LAD cohorts assayed by gene expression, DNA copy number or gene mutation sequencing were acquired [2], [6], [8], [11], [17], [18], [25]. The details of cohort composition and complete methodology including microarray processing are presented in Tables S1, S2 and S3.

**Figure 1:**
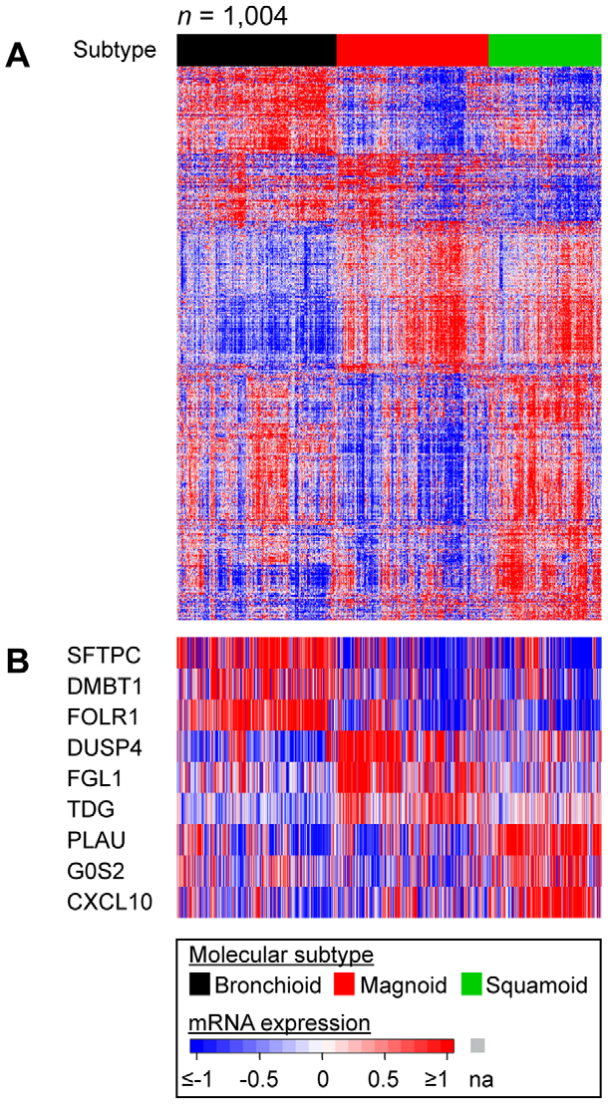
Lung adenocarcinoma molecular subtype expression characteristics. LAD subtype gene expression is displayed for all cohorts (Bhattacharjee et al., Chitale et al., Ding et al., Shedden et al., Tomida et al., UNC, Zhu et al.) in which columns are tumors and rows are genes and shading indicates gene expression level (A). Exemplar genes are displayed separately for visualization ease (B).

**Table 1:** : Subtype clinical and morphological characteristics.

Characteristic Clinical	*n*	Statistic	Bronchioid	Magnoid	Squamoid	
*unique patients*	1,004	total	370	360	274	
*Sex*	989	% female	62	44	50	*
*Age*	771	median	65	65	64	
*nonsmoker*	860	%	30	6	18	*
*pack years*	456	median	15	40	36	*
*Stage*	954	% I	75	59	54	*
		% II	14	24	20	
		% III	10	16	23	
		% IV	1	1	2	
*Grade*	794	% well	32	11	1	*
		% mod	53	49	38	
		% poor	15	40	61	
**Morphological**						
*features*						
*adenosquamous*	230	%	4	3	18	+
*bronchioloalveolar*	358	%	31	13	13	*
*necrosis*	87	%	4	52	43	*
*invasion*	87	%	71	94	100	+
*lymphocytes*	89	%	15	9	30	
*percentages*						
*papillary*	41	median	51	31	0	+
*acinar*	41	median	40	30	20	+
*solid*	40	median	0	3	80	*
*fibrosis*	50	median	20	10	3	
*tumor cellularity*	283	median	80	80	80	

Patient characteristics are summarized over all cohorts. The number of patients having a characteristic (*n*) varies because all cohorts do not include all characteristics. The percent statistics indicate the percent of tumors in a subtype having a given characteristic, e.g. 75% of the tumors in the Bronchioid subtype are stage I. The percentage characteristics refer to percentage values given to individual tumors, and subtype median values are presented, e.g., the median papillary percentage of tumors in the Bronchioid subtype is 51. Categorical and continuous variables were compared by Fisher's exact tests and Kruskal-Wallis tests, respectively (*: two-sided *P*<0.001; +: two-sided *P*<0.05). Cohort stratification was evaluated and did not change these results' significance.

**Table 2:** : Molecular subtypes compared by gene sequence mutations.

Discovery cohorts (Bhattacharjee et al., Chitale et al., Ding et al., Tomida et al.)						
	Bronchioid	Magnoid	Squamoid	*P*′	q-value	Subtype with greatest mutation frequency
*EGFR*	**37%**	9%	18%	4.96×10^−10^	3.47×10^−9^	Bronchioid
*KRAS*	16%	**30%**	18%	0.00410	0.0113	Magnoid
*TP53*	22%	**40%**	33%	0.00484	0.0113	Magnoid
*STK11*	13%	**22%**	8%	0.0691	0.121	Magnoid
*LRP1B*	0%	17%	29%	0.101	0.125	-
*BRAF*	5%	0%	2%	0.107	0.125	-
*PTEN*	3%	3%	8%	0.344	0.344	-

Validation cohort (UNC)					
	Bronchioid	Magnoid	Squamoid	*P*″	q-value
*EGFR*	**24%**	7%	6%	0.0293	0.0293
*KRAS*	5%	**37%**	15%	4.48×10^−4^	7.95×10^−4^
*TP53*	10%	**58%**	15%	6.40×10^−7^	2.56×10^−6^
*STK11*	3%	**29%**	0%	5.96×10^−4^	7.95×10^−4^

Percentages are the proportion of tumors having a mutation within a subtype, e.g. 37% of Bronchioid tumors have an *EGFR* mutation in the discovery cohorts. Corresponding numbers of patients with gene mutations are presented in Table S5. The association of subtype and mutations in the discovery cohorts were compared by Fisher's exact tests on three subtypes (two-sided *P*′). For significantly associated genes (*P*′<0.10), the subtype with the greatest mutation frequency was identified and is listed. In the validation cohort, Fisher's exact tests evaluated the null hypothesis that each gene's subtype with greatest mutation frequency had not greater mutation frequency than the other subtypes (one-sided *P*″). Benjamini-Hochberg false discovery rates (q-values) are also displayed for the discovery and validation cohort.

### Tumor collection and genetic assays

Retrospective macrodissected lung adenocarcinomas from patients receiving surgery for curative intent were collected at the University of North Carolina at Chapel Hill (UNC) under a waiver of consent by the UNC Biomedical Institutional Review Board approved protocol #07-0120. Gene expression was measured by Agilent 44 K microarrays. DNA copy number was measured by Affymetrix 250 K Sty and SNP6 microarrays. DNA methylation was measured by the MSNP microarray assay [26]. DNA from *EGFR*, *KRAS*, *STK11* and *TP53* exons were sequenced by ABI sequencers (Table S4). Mutations were non-synonymous or splice site differences compared to reference sequence [27]. These data were publicly deposited (http://www.ncbi.nlm.nih.gov/geo/query/acc.cgi?acc=GSE36471).

**Figure 2:**
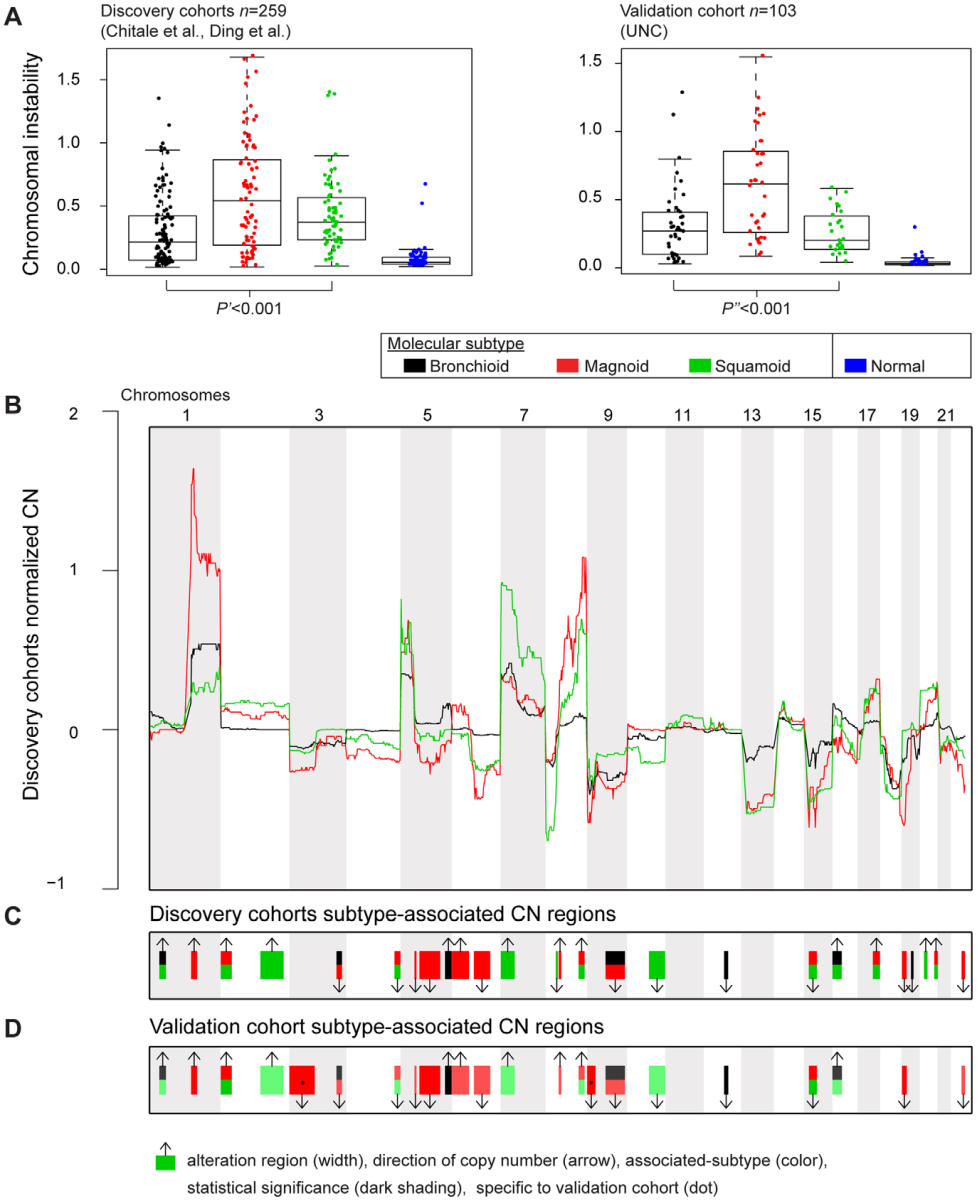
Molecular subtypes compared by chromosomal instability and regional DNA copy number. Chromosomal instability (CIN) grouped by molecular subtypes is displayed for discovery cohorts with CN (Chitale et al., Ding et al.) and the validation cohort (UNC) (A). Subtype CIN was compared by a Kruskal-Wallis test on three subtypes of the discovery cohorts (two-sided *P*′). A Wilcoxon rank-sum test evaluated the null hypothesis that Magnoid had not greater CIN than other subtypes in the validation cohort (one-sided *P″*). Normal lung specimens' CIN are shown for reference. The subtype regional DNA copy number (CN) medians of the discovery cohort are displayed (B). CN values below zero indicate copy number deletion, at zero indicate normal copy number, and above zero indicate copy number amplification. Subtype-associated CN regions from the discovery cohorts are displayed (C), in which the subtype with greatest absolute copy number is indicated by colored rectangle. For independent confirmation, these regions and associated-subtypes were tested in the validation cohort, results of which are displayed (D). For example in discovery cohorts, CN of 1q21–23 was significantly different among subtypes with Magnoid having the greatest CN (C), and in the validation cohort, Magnoid had significantly greater absolute CN compared to other subtypes (D). Afterwards, subtypes were compared by all regions in the validation cohort and new regions are marked by a dot (D).

**Figure 3:**
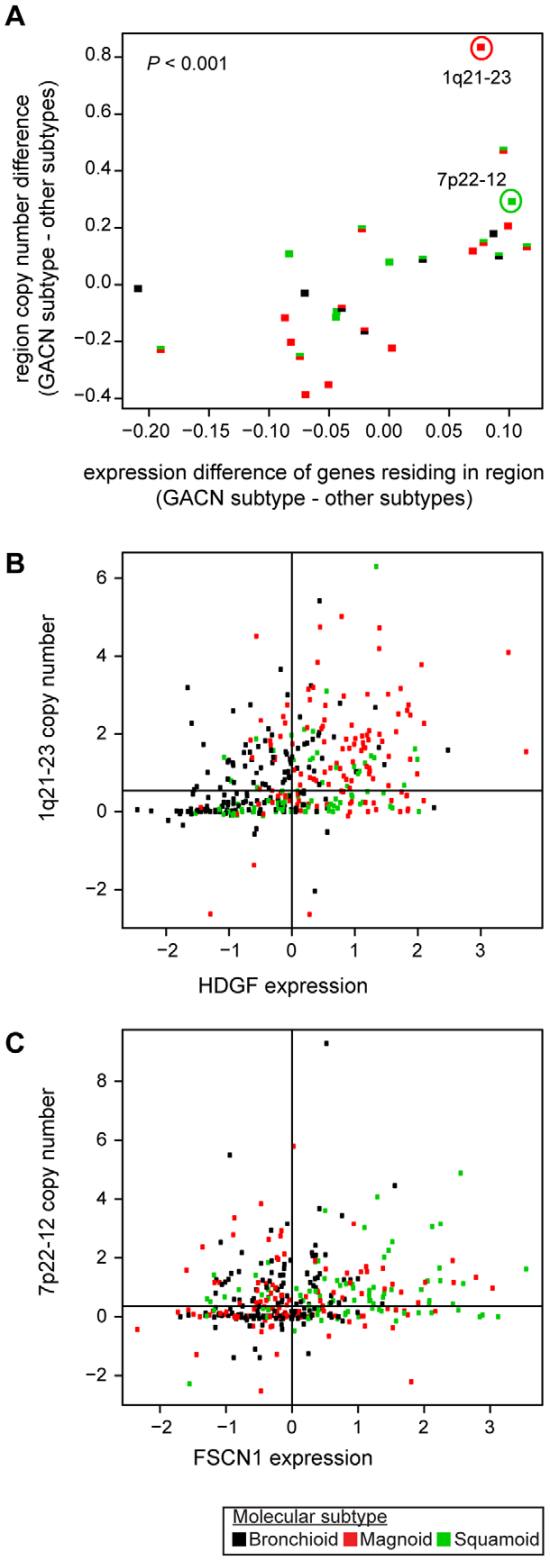
Coordination of DNA copy number and gene expression among subtypes. Each point represents one of the 26 subtype-associated copy number (CN) regions, which are colored by the subtype having the greatest absolute copy number (GACN). The vertical axis is the difference in median CN between the GACN subtype and other subtypes. For the genes in each region, the differences in median expression between the GACN subtype and other subtypes were calculated and the median of these differences is the value on the horizontal axis. The association of CN difference and gene expression difference across these regions was compared by a Spearman correlation test (two-sided *P*). Two example DNA regions are circled. Region 1q21–23 had GACN in Magnoid. Hepatoma-derived growth factor (*HDGF*) was one of the most Magnoid overexpressed genes in this region. CN and gene expression for *HDGF* is displayed in which each point is one tumor (B). Region 7p22-12 had GACN in Squamoid. Fascin (*FSCN1*) was the most Squamoid overexpressed gene in this region (C). For reference, black lines in (B, C) indicate median gene expression and DNA CN. LAD tumors with copy number and expression arrays from all cohorts were used (*n* = 362).

**Figure 4:**
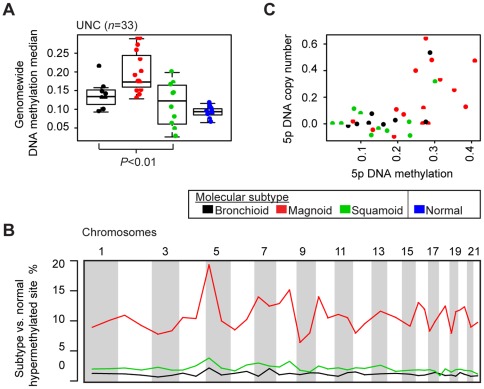
Molecular subtypes compared by DNA methylation. Genomewide DNA methylation among the three LAD subtypes were compared by a Kruskal-Wallis test (two-sided *P*) in all cohorts with methylation (UNC *n* = 33) (A). Normal lung specimens are shown for reference. Regional variation in DNA methylation is displayed (B). For each chromosome arm, the proportion of sites hypermethylated in a subtype with respect to normal lung is plotted. Chromosome arms with at least 4 methylation sites are displayed. Tumors' DNA methylation and DNA copy number values for chromosome 5 p are displayed (C).

### Molecular subtype assignment

In order to derive standardized molecular subtype assignments for all LAD tumors in this study, molecular subtypes were detected using gene expression as previously described [14] using ConsensusClusterPlus [28] and the largest published cohort, Shedden et al. A nearest centroid subtype predictor [29] utilizing 506 genes was trained on the Shedden et al. cohort and applied to all LAD tumors. The subtype predictor centroids were then publically posted (http://cancer.unc.edu/nhayes/publications/adenocarcinoma.2012/).

**Figure 5:**
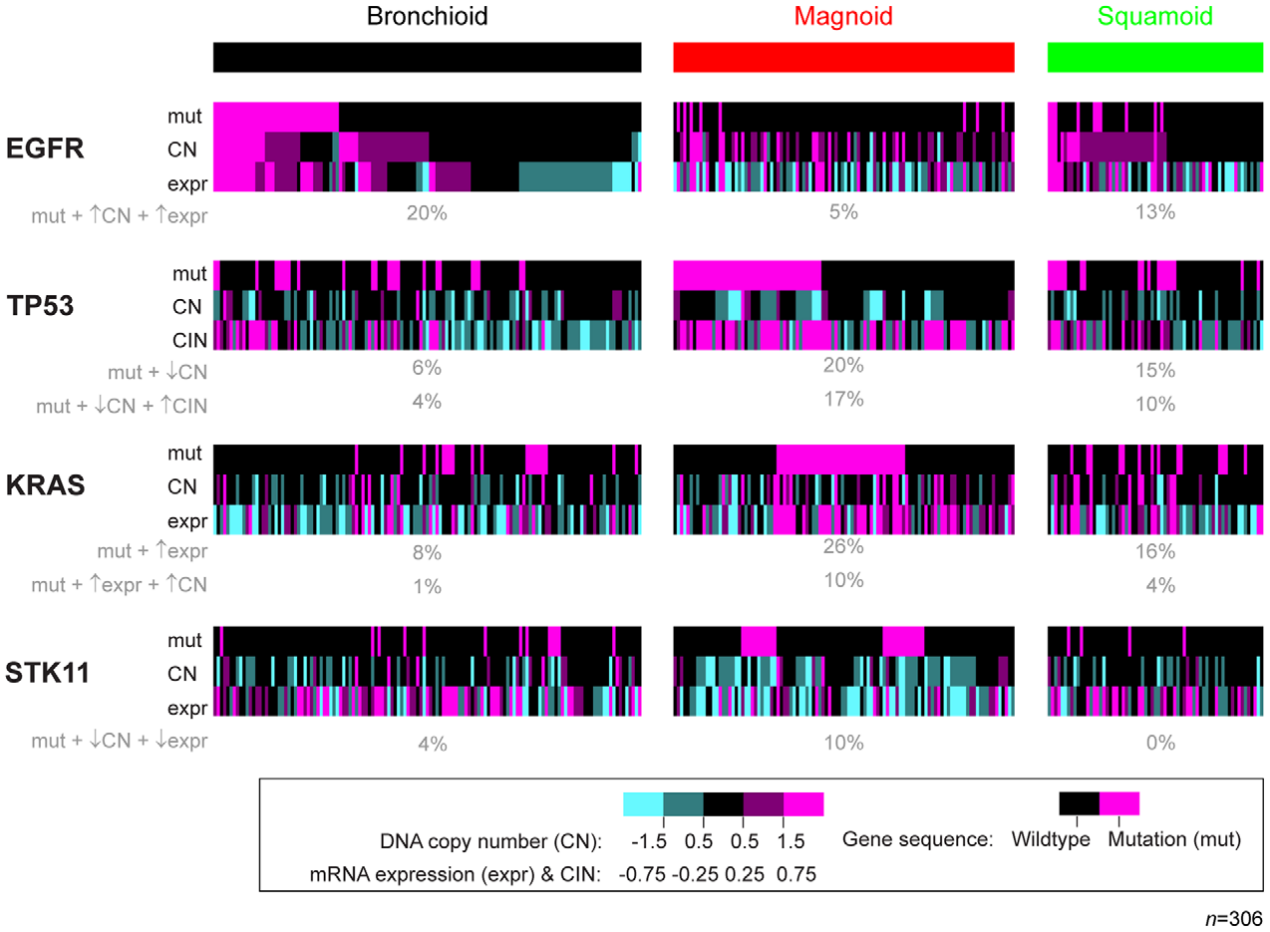
Integrated gene alterations compared among molecular subtypes. Tumors are depicted as columns and genetic features as rows from cohorts with CN and gene sequencing (Chitale et al., Ding et al., UNC). Gene CN's were defined by the gene's genomic position. The percentages of tumors within a subtype having a given integrated combination of alterations are displayed in grey. Fisher's exact tests on each integrated combination and subtype were statistically significant (*P*<0.01).

**Figure 6:**
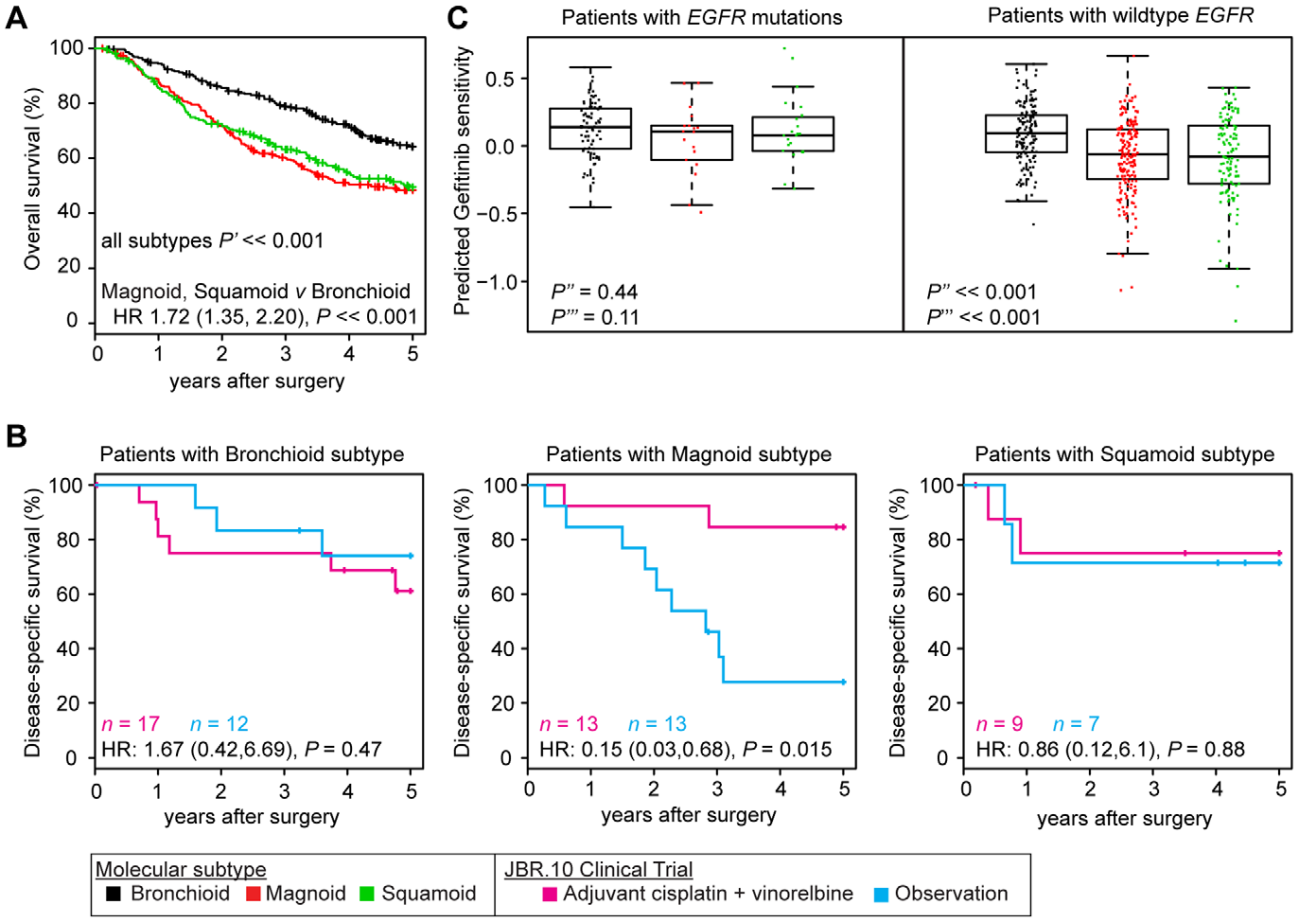
Patient outcomes compared among molecular subtypes. Overall survival is displayed for patients from cohorts with survival follow-up (Bhattacharjee et al., Chitale et al., Shedden et al., Tomida et al., UNC, and Zhu et al.; *n* = 807) (A). Overall survival among the three subtypes was compared by a log-rank test (two-sided *P*′). Patient disease-specific-survival from the Zhu et al. JBR.10 trial 25] is displayed by treatment type, for each subtype (B). Hazard ratios (HR) compare treatment to observation. Gefitinib sensitivity scores, derived from a cell line expression signature, are displayed for all patients with *EGFR* mutation status (Bhattacharjee et al., Ding et al., Chitale et al., Tomida et al., UNC; *n* = 561) (C). Predicted gefitinib sensitivities were compared by Kruskal-Wallis tests among the three subtypes in the *EGFR* mutation group and separately for the *EGFR* wild type group (two-sided *P*″). Wilcoxon rank-sum tests evaluated the hypothesis that Bronchioid tumors have not greater scores than Squamoid and Magnoid tumors within each *EGFR* mutation group separately (one-sided *P*″′).

### Survival analysis

Patients with less than one month of follow-up time were assumed to have surgical complications and removed from overall survival analyses (Bhattacharjee et al., 2 patients; Shedden et al., 3 patients; UNC, 3 patients, Zhu et al., 1 patient). Because some patients were common between the Shedden et al. and Chitale et al. cohorts, survival and redundant clinical data were removed from the Chitale et al. cohort. Patient disease-specific-survival was compared by subtype and treatment in the Zhu et al. cohort [25]. Survival outcomes were estimated by the Kaplan-Meier method and hazard ratios were estimated by Cox proportional hazards.

### DNA copy number (CN) analysis

Chromosomal instability (CIN) is a per tumor genomewide measure of CN instability. CIN was defined as the median of chromosome arm absolute copy numbers within a tumor, similar to published studies [30], [31]. Large values represent high CIN. DiNAMIC [32] identified regions of recurrent CN alterations in published cohorts. Tumors were assigned their median CN across probes within these regions. Region CNs were compared among the subtypes by Kruskal-Wallis tests using the discovery cohorts. For each region with significantly different CN (Benjamini-Hochberg adjusted *P*<0.05), the subtype with greatest absolute copy number (GACN) was identified by taking the subtype partition (Bronchoid versus Magnoid and Squamoid; Magnoid versus Bronchioid and Squamoid; or Squamoid versus Bronchioid and Magnoid) with the greatest absolute difference in median CN, and identifying the group farthest from zero (which is the normal CN value). These regions and GACN subtypes were compared in the validation cohort using Wilcoxon rank-sum tests with the null hypothesis that absolute CN is not greater in the GACN subtype versus other subtypes with significant results having *P*<0.05. Afterwards, all regions were compared in the validation cohort to detect possible subtype associations not found in the discovery cohort (Kruskal-Wallis tests, Benjamini-Hochberg adjusted *P*<0.05). Human genome assembly hg 18 coordinates were used for probe locations.

### Methylation-SNP (MSNP) analysis

Methylation was measured by comparing the abundance of DNA digested by methylation-sensitive *HpaII* to *HpaII*-undigested DNA by Affymetrix 250 K Sty microarrays [26]. The arrays had 57,566 *HpaII* (methylation) sites and 169,804 control sites. Methylation was quantified by percent change relative to the undigested DNA abundance, (undigested – *HpaII*) / undigested. Increasing values indicate increasing methylation.

### Integrated genetic analyses

Several integrated analyses between molecular subtype and one or more genetic variables were performed. For each particular integrated analysis, all tumors with the necessary assays were selected, normalized within their cohort, pooled and analyzed together (details listed in Table S2).

### Gefitinib sensitivity prediction

A published expression signature that predicted gefitinib sensitivity based on baseline gene expression in non-small cell lung cancer cell lines [33] was used to assign predicted gefitinib sensitivity scores to tumors. The published gefitinib overexpressed genes and underexpressed genes were combined to define a gefitinib sensitivity score for each tumor: 
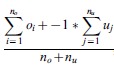
, where *o* and *u* represent expression of the overexpressed and underexpressed genes, respectively, and *n* represents the gene total. Increasing scores indicate increasing sensitivity.

Computational procedures were executed using R (http://www.r-project.org) and Bioconductor libraries (http://www.bioconductor.org ) unless otherwise specified.

## Results

### Molecular subtype characteristics

In order to compare genomic alterations among molecular subtypes, tumors were assigned to a lung adenocarcinoma (LAD) molecular subtype (Bronchioid, Squamoid, or Magnoid) using published methods (Fig. S1, S2, 1A). Consistent with earlier reports [11], [13], [14], the subtypes overexpressed genes involved in distinctive biological processes including: Bronchioid – excretion genes, asthma genes, and surfactants (*SFTPB*, *SFTPC*, *SFTPD*); Magnoid – DNA repair genes, such as thymine-DNA glycosylase (*TDG*); Squamoid – defense response genes, such as chemokine ligand 10 (*CXCL10*) (Fig. 1B). Confirming and extending earlier studies [11], [13], [14], [16], the subtypes had very different clinical profiles (Table 1). Bronchioid had the most females, nonsmokers, early stage tumors, and low grade tumors, the greatest acinar content, the least necrosis, and the least invasion. Squamoid had the most high grade tumors, the greatest solid content, and the lowest papillary content. Magnoid had the most smokers and the heaviest smokers by pack years. Bronchioloalveolar and adenosquamous features were most prevalent in the Bronchioid and Squamoid subtypes, respectively, consistent with their expression similarities to these histological subclasses (Fig. S2).

### Distinctive gene mutations of the molecular subtypes

To determine if the molecular subtypes co-occurred with different mutant genes, the discovery cohorts (published cohorts with gene mutation status: Bhattacharjee et al., Chitale et al., Ding et al., Tomida et al.) were pooled and filtered for genes in which five or more patients had mutations (*BRAF*, *EGFR*, *KRAS*, *LRP1B*, *PTEN*, *STK11* and *TP53*). Of these, four mutant genes were significantly associated with molecular subtype (two-sided *P*<0.10, Table 2 and Table S5). Bronchioid had the greatest *EGFR* mutation frequency, while Magnoid had the greatest mutation frequencies in *TP53*, *KRAS* and *STK11*. To independently validate these subtype and mutant gene associations, these four genes were sequenced in the UNC cohort. Again, Bronchioid had a significantly greater *EGFR* mutation frequency than the other subtypes and Magnoid had greater mutation frequencies of *TP53*, *KRAS* and *STK11* than other subtypes (Table 2 and Table S5). These results convincingly demonstrated that the LAD molecular subtypes have distinct mutation frequencies of four genes and that the subtypes tend to have different genes mutated.

To evaluate if the LAD molecular subtypes also have different genomewide mutation rates, a large set of rarely mutated genes (*n*  = 623) from the Ding et al. cohort was used to calculate genomewide nonsynonymous mutation rates for each tumor. Mutation rates were significantly different among the subtypes with increasing mutation rates observed in Bronchioid, Squamoid and Magnoid (Table S6).

### Contrasting chromosomal instability and regional DNA copy number alterations by molecular subtype

To detect DNA copy number (CN) differences among the subtypes, the discovery cohorts (published cohorts with DNA CN: Chitale et al., Ding et al.) were pooled and analyzed. A chromosomal instability (CIN) statistic was calculated for each tumor that represents the average absolute magnitude of CN alterations over the genome. CIN values of zero indicate a completely normal genome and increasing positive values indicate a genome with an increasing degree of copy number alteration. CIN was significantly different among the subtypes, with Magnoid having the highest CIN (Fig. 2A). Again in the UNC cohort, Magnoid had significantly greater CIN than the other subtypes; therefore, Magnoid's high CIN was independently validated (Fig. 2A).

Subtype CN profiles revealed regional CN differences among the subtypes in the discovery cohorts (Fig. 2B). To identify standardized regions in which to compare CN, the discovery cohorts were queried for statistically altered regions without regard to subtype, yielding 39 regions (Table S7). Subtypes were compared by median CN within these regions in the discovery cohorts. 26 of the 39 regions had statistically different CN among the subtypes (Fig. 2C). For each region, the subtype having the greatest absolute copy number (GACN) was identified. For example, Magnoid had GACN in region 1q21–23 (Fig. 2B,C). For independent validation, the 26 regions and corresponding GACN subtypes were compared in the UNC cohort. Remarkably, in 21 regions, the subtype with GACN in the discovery cohorts had greater absolute CN than the other subtypes in the UNC cohort, reaching statistical significance in 7 of the 21 regions (Fig. 2D). Afterwards, all regions were compared directly in the validation cohort, yielding only two new subtype-associated regions (3p26-12 and 9p24-21) indicating that the majority of subtype-associated regions are not dependent on cohort or platform. Overall, the subtype-associated regional CN alterations were independently validated.

CN alterations are known to affect expression of genes residing within altered regions across lung cancer [4], suggesting that the subtype-specific regional CN may correlate with subtype-specific regional gene expression. To test this hypothesis, the difference in CN of the 26 regions and the difference in median expression of genes residing within the regions were calculated between the corresponding GACN subtype and other subtypes –(Fig. 3). Regional CN difference and regional expression difference were significantly positively correlated. In other words, the subtype with the greatest amplification for a region also exhibited the greatest overexpression of genes in the region, and similarly for deletion and underexpression. Therefore, subtype-specific CN alterations likely contribute to causing the subtype-specific gene expression profiles.

### Divergence in DNA methylation among molecular subtypes

In addition to genetic alterations, the molecular subtypes may have different epigenetic modulations, namely DNA methylation alterations. Published LAD cohorts with gene expression and DNA methylation were not available, so subtype differences were detected directly in the UNC cohort. Over all methylation sites genomewide, the LAD subtypes had significantly different median methylation (Fig. 4A). Magnoid exhibited increased methylation (“hypermethylation”) compared to other subtypes and to normal lung.

Similar to the genomewide trend, the most differentially-methylated sites were hypermethylated in Magnoid compared to normal lung, followed by Squamoid and Bronchioid (Table S8). Interestingly, the genomic positions of sites hypermethylated in subtypes compared to normal lung showed regional variation with a peak at 5 p (Fig. 4B). CN and methylation at 5 p were positively related, with Magnoid tumors having the greatest values of both CN and methylation (Fig. 4C). *TERT* resides in 5 p and is affected by aberrant hypermethylation and deregulated expression in cervical cancer [34], suggesting a target in this region for Magnoid. Sites hypermethylated in normal lung compared to tumors were much rarer and between-subtype site differences were negligible and did not exhibit regional variation. Taken together, the main methylation alteration was genomewide hypermethylation in Magnoid.

### Integrated alterations among molecular subtypes

In addition to single alterations, the molecular subtypes possessed different genes affected by integrated alterations in the same tumors (Fig. 5). The Bronchioid subtype was distinctly enriched with tumors having integrated mutation, CN amplification and overexpression in *EGFR*. The Magnoid subtype had the greatest prevalence of tumors with integrated mutation and CN deletion in *TP53*. *TP53* expression was not consistently associated with *TP53* mutation. Interestingly, Magnoid *TP53* altered tumors also had frequent CIN, one of the consequences of mutant *TP53* [35]. So, *TP53* appears to be an important inactivated gene in Magnoid; however, Magnoid had additional integrated altered genes indicating that *TP53* was not its lone gene of importance. Tumors with integrated mutation, CN deletion and underexpression in *STK11* were most prevalent in the Magnoid subtype. The Magnoid subtype also contained the most tumors with integrated mutation, CN amplification and overexpression in *KRAS*. In sum, the subtypes distinctively contained tumors with integrated alterations in *EGFR*, *KRAS*, *STK11*, and *TP53*, suggesting these genes may be subtype-specific drivers in cancer development.

In addition to alterations targeting the same gene, concurrent mutant gene combinations were prominent among the subtypes. Focusing on tumors with an *EGFR* mutation, Bronchioids typically had *EGFR* mutations as a solitary mutation and rarely concurrently with *TP53* (Fig. S3A). Contrastingly, non-Bronchioid *EGFR* mutants often also had concurrent *TP53* mutations (Fig. S3A). Remarkably among tumors having a *TP53*, *STK11* or *KRAS* gene mutation, Magnoids frequently had two of these genes concurrently mutated in the same tumors [ (*TP53* plus *STK11*), (*TP53* plus *KRAS*) or (*KRAS* plus *STK11*)] while these combinations occurred much less frequently in other subtypes (Fig. S3B, C, D). Therefore, concurrent mutant gene combinations, in addition to single mutations, may be subtype-specific drivers in cancer development.

### Patient survival and therapy response vary by molecular subtypes

Patient overall survival was significantly different among the subtypes, with Bronchioid having the best outcome (Fig. 6A), as previously reported [14]. After controlling for all well-measured possible confounders including stage, grade and age, subtype significantly predicted overall survival.

Patient therapy response was retrospectively compared using the Zhu et al. clinical trial cohort that included tumor gene expression profiling prior to adjuvant treatment [25]. Patient disease-specific survival after treatment was markedly different among the subtypes (Fig. 6B). Specifically, only Magnoid patients exhibited clinically and statistically significant improved disease-specific survival compared to observation alone. In stark contrast, Bronchioid and Squamoid patients showed no benefit. Although the overall numbers were small, there was even a suggestion of an inferior outcome for Bronchioid patients treated with chemotherapy compared to observation. Possibly, Magnoid cancers are sensitive because of DNA repair defects, similar to basal-like breast cancers that are also sensitive to cisplatin [36] and also have *TP53* mutations [37] and high CIN [38].

Because of its specific association with *EGFR* alterations (mutation, amplification, overexpression and integrated alterations), Bronchioid tumors may have the greatest sensitivity to *EGFR* inhibitors. To our knowledge, there is no publically-available clinical trial cohort with patient anti-*EGFR* therapy response and gene expression. However, surrogate markers predictive of anti-*EGFR* therapy response were available, including a validated cell line expression signature of gefitinib sensitivity [33]. Tumors were assigned gefitinib sensitivity scores using this signature. As expected, *EGFR* mutant tumors had significantly greater predicted gefitinib sensitivity than *EGFR* wildtype tumors (Fig. 6C, Wilcoxon rank-sum test, one-sided *P*<<0.001). Additionally among *EGFR* tumors, Bronchioids had greater average sensitivity scores compared to Magnoids and Squamoids. Strikingly among *EGFR* wildtype tumors, Bronchioids had significantly greater average sensitivity than Squamoids and Magnoids and also had sensitivity scores similar to those of non-Bronchioid *EGFR* mutant tumors. This suggests that patients with wild type *EGFR* who benefit from *EGFR* inhibitory therapy are more likely to be in the Bronchioid subtype than other subtypes. Moreover, this suggests that molecular subtype may have independent predictive value for therapy response. Although the difference in means is statistically significant, there were small proportions of Bronchioids with low sensitivity and non-Bronchioids with high sensitivity indicating that subtype does not discriminate every single case. Future studies of expression profiling and therapy response on the same patients would be able to evaluate this directly.

## Discussion

Data presented herein have shown that lung adenocarcinomas in the intrinsic molecular subtypes have significantly different alterations in gene sequence mutations, chromosomal instability, regional DNA copy number, DNA methylation, and integrated combinations. We strengthen the evidence for subtype-alteration associations by *a priori* hypothesis testing in a previously-uncharacterized cohort from our institution, which provided substantial confidence that these associations are robust. Our results indicate that the intrinsic, naturally-occurring molecular subtypes are not only a gene expression phenomenon but also a representation of different variants of LAD disease defined by different genomic alterations.

Whether in clinical management or in laboratory models, LAD is primarily classified by morphology, mutations, or clinical characteristics. The LAD intrinsic molecular subtypes capture many clinically-relevant phenotypes from these separate classifications. Bronchioid is represented by patients who are female and nonsmoking, who have a superior survival outcome, and who present with well-differentiated, bronchioloalveolar morphology, early stage and *EGFR* mutated cancers [6], [8], [14], [16], [19], [21], [39]. High smoking exposure, poor survival outcome and late-stage presenting patients are common in the other two subtypes: Magnoid and Squamoid. Magnoid has a high prevalence of patients who are male and have *KRAS*, *TP53* or *STK11* alterations [6], [19]. Squamoid includes patients who present with poorly differentiated and solid morphology cancers [6], [16], [40].

This comprehensive genomic analysis provides significant insights into subtype-specific alterations. For example not only does Bronchioid have the most *EGFR* mutations, this subtype also has the most patients with integrated mutation, amplification and overexpression of *EGFR*. Although this combination has been observed [2], [6], [41], this is the first evidence that it predominantly occurs in one molecular subtype. Development of the Bronchioid subtype seems to be uniquely dependent on mutant *EGFR*, while the rare *EGFR* mutants in other subtypes usually have concurrent *TP53* mutation. Finally, Bronchioid had the most *BRAF* mutants, suggesting a second less common Bronchioid driver, although this mutation is too rare to confirm by our analysis.

In addition to having the most *TP53*, *STK11* and *KRAS* mutations, Magnoid tumors also have severe genomic alterations including the greatest CIN, the most regional CN alterations, DNA hypermethylation, and the greatest genomewide mutation rate. Magnoid's overexpression of DNA repair genes suggests that these tumors are actively repairing their heavily damaged genome, possibly in response to these patients' heavy smoke exposure. Magnoid has the most genes with concurrent alterations in the same patients (*TP53*, *STK11*, *KRAS*), unlike Bronchioid which typically had *EGFR* as its sole sequence mutation as discussed above. This again suggests that excessive DNA damage occurred in these tumors, perhaps due to smoking exposure, and has driven multiple gene mutations. Magnoid's concurrency of *TP53* alterations and high CIN is a novel association within an LAD patient subgroup and could be explained by *TP53* alteration inducing high CIN, similar to recently reported cell culture studies [35]. Finally, the Magnoid subtype exhibited increased hypermethylation, a phenomenon similar to the CpG-Island-Methylator-Phenotype (“CIMP”) observed in other cancers [42], [43].

Lastly, the Squamoid subtype displayed the fewest distinctive alterations that included only regional CN alterations. Adenosquamous features were most prevalent in Squamoid, which is the first association with a molecular subtype to date. Squamoid had the most *PTEN* mutations and loss of its locus, 10q22–q26, suggesting this may be a Squamoid-specific driver; however, *PTEN* mutation was too rare to confirm by our analysis. Interestingly, the Squamoid subtype, which presents in patients as a poorly differentiated solid morphology cancer and predicts poor survival, had the fewest distinctive genomic alterations.

This study reports associations using retrospective cohorts and determination of causation is not possible. However, because cancers stratified by the molecular subtypes have different genomic alterations and because genomic alterations cause cancer [44], we infer that cancers stratified by the molecular subtypes have arisen by different genomic alterations, so called ‘molecular pathogenesis’. But beyond subtype and genomic alterations co-associating in cancers, is there more to the nature of this relationship? We offer several possible explanations. One model is that genomic alterations change a cancer's gene expression and that differences in genomic alterations directly cause the three molecular subtypes we observe. Supporting evidence is provided by mouse studies with activated cancer genes producing tumors with varied gene expression. However, it remains unlikely that alterations by themselves control cancer gene expression. Therefore, an alternative model is that subtypes and alterations are both caused by additional factors, such as the cancer's cell type of origin, patient behavior such as smoking, and/or patient germline sequence. Properties of a cancer's original cell type may promote specific genomic alterations due to physical mutability or the selective advantage that a specific mutation confers on a specific cell type. Considering that lung adenocarcinoma and lung squamous cell carcinoma have very different mutational profiles and are believed to originate from distinct cell types [19], differences in cell of origin seems to be a reasonable model. This model is also supported by observations of increased *EGFR* mutation prevalence in the terminal respiratory units of the lung compared to other areas [45]. We surmise that associations between subtypes and alterations can be explained by differences in cell type of origin that incur different alterations, promoted by different patient characteristics, which combined results in a different molecular subtype and patient outcome.

The LAD molecular subtypes and their associated alterations have clear translational significance. This study represents a second validation of the survival advantage for the Bronchioid subtype of LAD, a disease with few clinically implemented biomarkers. Additionally, we present data suggesting that molecular subtypes have relevance in predicting response both to cytotoxic chemotherapy and targeted *EGFR* inhibitory therapy beyond the established role of *EGFR* mutation status. These observations were derived from retrospective clinical data and surrogate response markers. Future prospective clinical trial and model systems studies are needed to confirm and more deeply describe the genomic basis of these findings.

In conclusion, we demonstrated that lung adenocarcinomas in different molecular subtypes have grossly distinct genomic alterations, clinical phenotypes, and clinical outcomes.

## Supporting Information

Figure S1 **Molecular subtype detection.** Unsupervised molecular subtype detection in the Shedden et al. cohort was conducted using the top 25% most variable genes, 3,045, using ConsensusClusterPlus [28]. The consensus matrix displays the result for a cluster total of 3. The consensus matrix is a symmetrical matrix of consensus values between pairs of tumors that is indicated by blue shading. High consensus corresponds to samples that always occur in the same cluster and is shaded dark blue. (A). Cumulative distributions of consensus are displayed for different cluster totals (*k*) (B). These were reviewed to determine the *k* that first approaches the maximum consensus, which indicates the most stable cluster total and indicates that further increases in *k* are insubstantially improving consensus [28], [48]. A large increase in consensus between *k = *2 and k = 3 was observed (B). *k = *3 was near the maximum consensus distribution achieved by greater cluster totals and the sizes of further clusters beyond 3 are small, as displayed in the item tracking plot in which tumors belonging to the same cluster are colored the same (C). Therefore, *k = 3* was determined to be the most stable clustering. All pairs of clusters in the *k = *3 clustering were significantly different by SigClust [49], which tests the hypothesis that two clusters are a result of chance alone (p-values in D). The new cluster segregated at *k = *4 is not significantly different from other clusters (pink shading in D), adding further support that the number of clusters is 3.
(PDF)

Figure S2 **Comparison of molecular subtypes to histological classes.** Our previously-published method of comparing subtypes to lung histological classes using centroids was followed [14]. Centroids for bronchioloalveolar (BAC), large cell carcinoma (LCC), and squamous cell carcinoma (SCC) were calculated by taking the gene-wise median as a centroid and gene-median centering these centroids within their cohort. UNC BAC samples are those adenocarcinomas exhibiting BAC features. Centroids for our previous published Hayes et al. subtypes were prepared using the Bhattacharjee et al. cohort as previously described [14]. This study's subtypes, from the Shedden et al. cohort, were described in Fig. S1. Centroid similarity was assessed by Pearson correlation coefficient using genes common among these cohorts and this study's subtype predictor. The correlation matrix depicts pairwise centroid similarities, according to the scale where dark purple represents strong gene expression similarity and dark gray represents strong gene expression dissimilarity (B). The centroids were clustered to determine subtype correspondences (agglomerative, average-linkage, hierarchical clustering) (A). Three groups were present in the clustering, indicated by the dendrogram and colored squares. In these groups, the same histological classes were grouped from the UNC and Takeuchi et al. cohorts (BAC, SCC, and LCC), which demonstrated cross-cohort consistency. Each group also had one member from the Hayes et al. and Shedden et al. cohorts, indicating that the subtypes detected in this study were consistent with the previously published Hayes et al. subtypes. Following Hayes et al., the Shedden et al. subtypes were named based on their unique similarities to lung histological classes as depicted in this centroid clustering, as follows: Shedden et al. Cluster 1–Bronchioid, Shedden et al. Cluster 2–Magnoid, Shedden et al. Cluster 3–Squamoid. The Magnoid and Squamoid names are reversed relative to Hayes et al. In Hayes et al., these two names were based on similarities, which had acknowledged subtle relationships and were based on a very small number (*n* = 5) of large cell carcinomas from one cohort [14]. Because this study has 29 large cell carcinomas from two independent cohorts, the subtype names derived by this study's results were used in this study. Besides this nomenclature difference, all significant results from Hayes et al. were consistent with this study.
(PDF)

Figure S3 **Gene sequence co-mutations.** Tumors are represented as columns and genes as rows. Tumors are from all cohorts with corresponding assays (Chitate et al., Ding et al., UNC). Percentages of tumors within a subtype having a particular gene mutation combination are listed. Associations of the integrated combination with subtype were tested by Fisher's exact tests (*P*).
(PDF)

Table S1 **Cohort data types and patient counts.** Patients common to Shedden et al. and Chitale et al. cohorts (*n* = 88) and those common to the Zhu et al. and Shedden et al. cohorts (*n* = 43) were counted once in the unique adenocarcinoma and lung cancer totals. Patients common to the Tomida et al. and Takeuchi et al. cohorts (*n* =  26) were counted once in the unique lung cancers total. Gene sequencing refers to the number of tumors with at least one gene sequenced.
(DOC)

Table S2 **Study design.** This table presents the order in which steps were followed and which datasets were used. ‘X’ indicates a cohort was used for a particular step. Separate platforms within a cohort were gene median centered separately and merged (*).
(DOC)

Table S3 **DNA copy number and methylation microarray processing.** The copy number (CN) and methylation microarray processing steps for each cohort are listed. ‘X’ indicates the step was followed. Affymetrix SNP arrays were processed by CRMAv2 [46]. The Affymetrix SNP6 microarrays were subjected to an outlier probe removal method to remove nonhybridizations similar to published methods [47]. Common probe locations were calculated by taking the median of probe CN values every 2 megabases.
(DOC)

Table S4 **Validation cohort gene sequencing regions.** These regions were sequenced in the UNC cohort by Polymorphic, Inc (Almeda CA) on ABI 3730XL DNA sequencers. Coordinates are from human genome assembly hg 18. Regions include some flanking intronic sequence.
(DOC)

Table S5 **Subtype gene sequence mutation counts.** Numbers of patients with mutant or not mutant (wild type) genes are listed by subtype. These numbers correspond to the percentages in Table 2. Patient counts vary by gene because not all cohorts sequenced all tumors for all genes.
(DOC)

Table S6 **Subtype genomewide mutation rates.** Non-synonymous genome wide rates were calculated for each tumor by diving the number of non-synonymous mutations by the number of nucleotides sequenced. Mutation rates were significantly different (Kruskal-Wallis test two-sided *P*<0.01). Confidence intervals were calculated by 1,000 bootstrap replicates.
(DOCX)

Table S7 **Regions of recurrent DNA copy number amplifications and deletions.** Regions of recurrent DNA copy number amplifications and deletions were calculated by DiNAMIC 32] (*P*<0.001). CN amplifications and deletions are indicated by 1 and –1, respectively. For each region, marker refers to the point of the most extreme CN, and left and right refer to the region's boundaries. Positions are hg 18 genomic coordinates.
(DOC)

Table S8 **Differentially methylated site totals.** Cells contain the number of sites with significantly greater methylation in the row class compared to the column class. Methylation was compared between classes by Wilcoxon rank-sum tests that evaluated the null hypothesis of not greater methylation in the row class (Benjamini-Hochberg adjusted *P*<0.05). For example, 11,720 sites had significantly greater methylation in Magnoid tumors compared to normal lung. Squamoid and Bronchioid hypermethylated sites compared to normal lung were almost completely contained in the Magnoid versus normal sites (83% and 88%, respectively).
(DOC)
