# Supplementary material for: Differential Pathogenesis of Lung Adenocarcinoma Subtypes Involving Sequence Mutations, Copy Number, Chromosomal Instability, and Methylation
Source: PLoS One. 2012 May 10;7(5):e36530. doi: 10.1371/journal.pone.0036530 (PMC3349715; doi:10.1371/journal.pone.0036530)
Supplement: Figure S1 — Molecular subtype detection. Unsupervised molecular subtype detection in the Shedden et al. cohort was conducted using the top 25% most variable genes, 3,045, using ConsensusClusterPlus [28]. The consensus matrix displays the result for a cluster total of 3. The consensus matrix is a symmetrical matrix of consensus values between pairs of tumors that is indicated by blue shading. High consensus corresponds to samples that always occur in the same cluster and is shaded dark blue. (A). Cumulative distributions of consensus are displayed for different cluster totals (k) (B). These were reviewed to determine the k that first approaches the maximum consensus, which indicates the most stable cluster total and indicates that further increases in k are insubstantially improving consensus [28], [48]. A large increase in consensus between k = 2 and k = 3 was observed (B). k = 3 was near the maximum consensus distribution achieved by greater cluster totals and the sizes of further clusters beyond 3 are small, as displayed in the item tracking plot in which tumors belonging to the same cluster are colored the same (C). Therefore, k = 3 was determined to be the most stable clustering. All pairs of clusters in the k = 3 clustering were significantly different by SigClust [49], which tests the hypothesis that two clusters are a result of chance alone (p-values in D). The new cluster segregated at k = 4 is not significantly different from other clusters (pink shading in D), adding further support that the number of clusters is 3. (PDF) [file pone.0036530.s001.pdf]

A Consensus matrix (ConsensusClusterPlus)

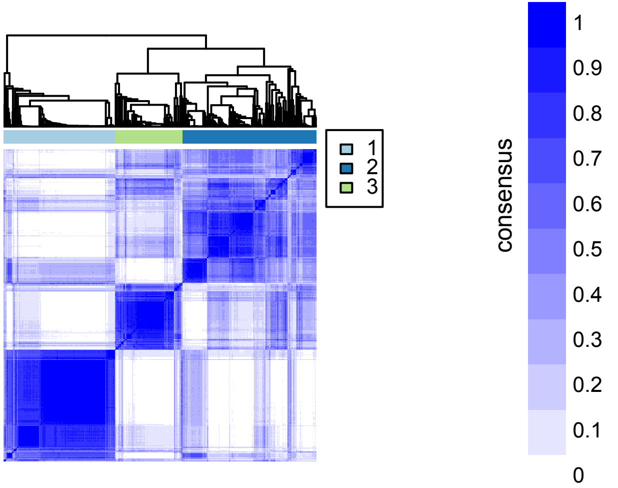

B Consensus cumulative distribution

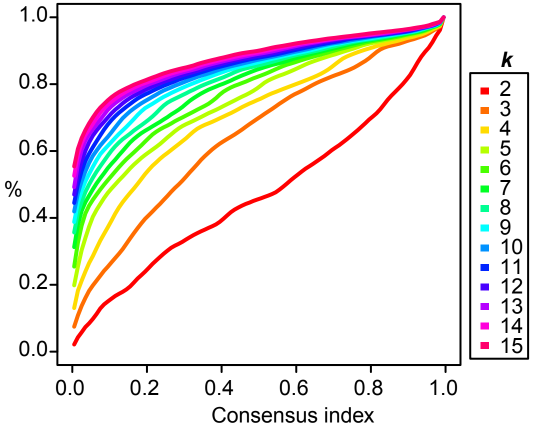

C Item tracking plot

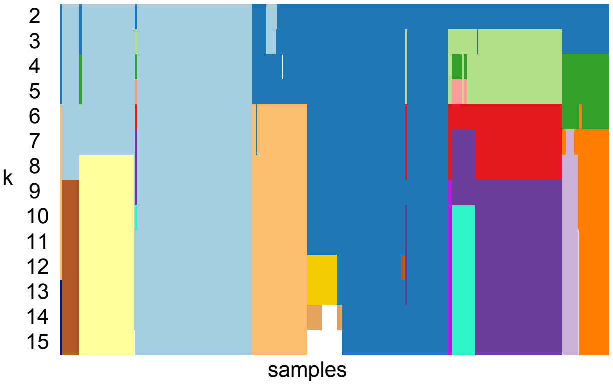

D Cluster significance testing (SigClust)

***k*=3**		cluster	
		1	2
cluster	1		
	2	2.78E-15	
	3	3.76E-16	7.86E-06

***k*=4**		cluster		
		1	2	3
cluster	1			
	2	2.80E-18		
	3	2.92E-17	1.26E-08	
	4	0.479	0.197	1.85E-06
