# Supplementary figures and images for: Differential Pathogenesis of Lung Adenocarcinoma Subtypes Involving Sequence Mutations, Copy Number, Chromosomal Instability, and Methylation
Source: PLoS One. 2012 May 10;7(5):e36530. doi: 10.1371/journal.pone.0036530 (PMC3349715; doi:10.1371/journal.pone.0036530)

A

B

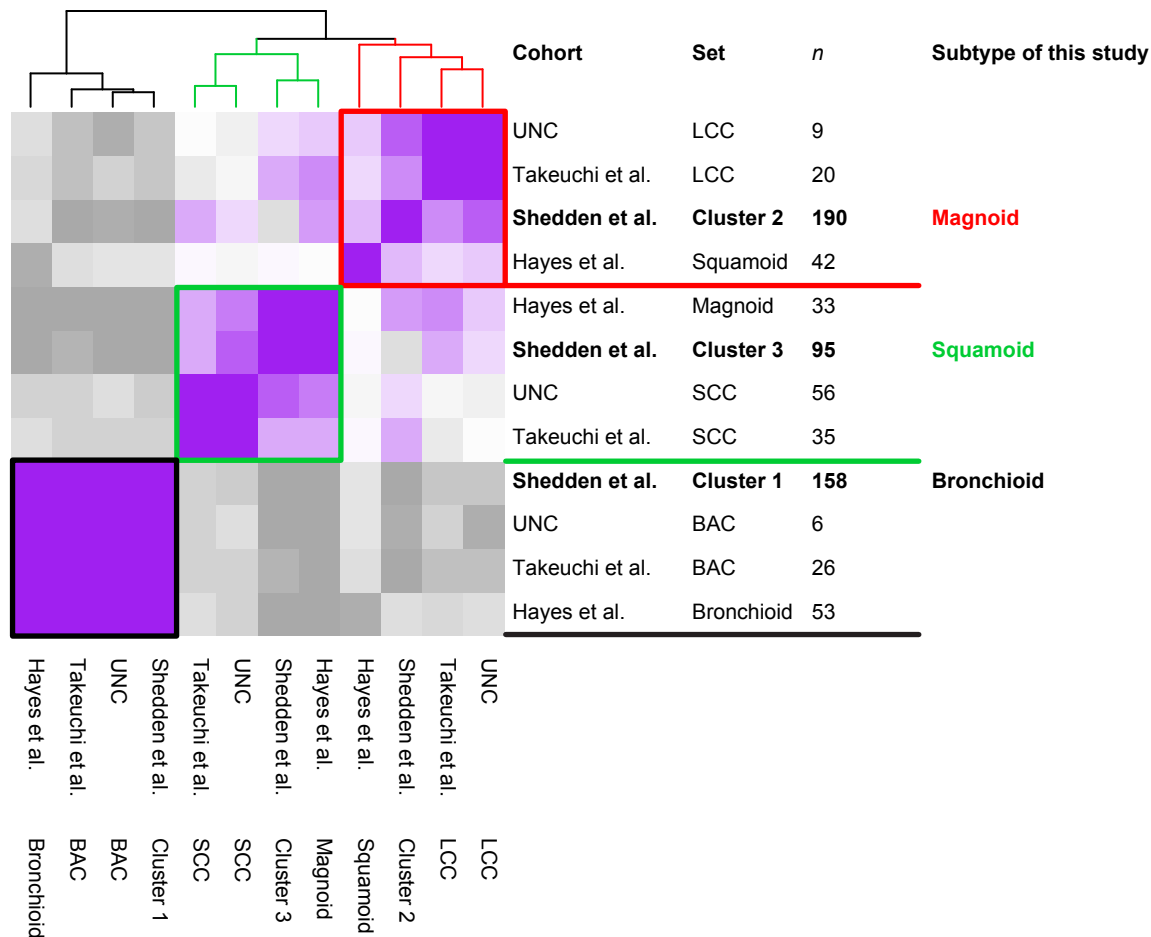

1- Pearson correlation coefficient  
 Pearson correlation coefficient

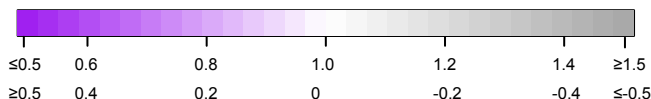

Supplement: Figure S2 — Comparison of molecular subtypes to histological classes. Our previously-published method of comparing subtypes to lung histological classes using centroids was followed [14]. Centroids for bronchioloalveolar (BAC), large cell carcinoma (LCC), and squamous cell carcinoma (SCC) were calculated by taking the gene-wise median as a centroid and gene-median centering these centroids within their cohort. UNC BAC samples are those adenocarcinomas exhibiting BAC features. Centroids for our previous published Hayes et al. subtypes were prepared using the Bhattacharjee et al. cohort as previously described [14]. This study's subtypes, from the Shedden et al. cohort, were described in Fig. S1. Centroid similarity was assessed by Pearson correlation coefficient using genes common among these cohorts and this study's subtype predictor. The correlation matrix depicts pairwise centroid similarities, according to the scale where dark purple represents strong gene expression similarity and dark gray represents strong gene expression dissimilarity (B). The centroids were clustered to determine subtype correspondences (agglomerative, average-linkage, hierarchical clustering) (A). Three groups were present in the clustering, indicated by the dendrogram and colored squares. In these groups, the same histological classes were grouped from the UNC and Takeuchi et al. cohorts (BAC, SCC, and LCC), which demonstrated cross-cohort consistency. Each group also had one member from the Hayes et al. and Shedden et al. cohorts, indicating that the subtypes detected in this study were consistent with the previously published Hayes et al. subtypes. Following Hayes et al., the Shedden et al. subtypes were named based on their unique similarities to lung histological classes as depicted in this centroid clustering, as follows: Shedden et al. Cluster 1–Bronchioid, Shedden et al. Cluster 2–Magnoid, Shedden et al. Cluster 3–Squamoid. The Magnoid and Squamoid names are reversed relative to Hayes et al. In [file pone.0036530.s002.pdf]

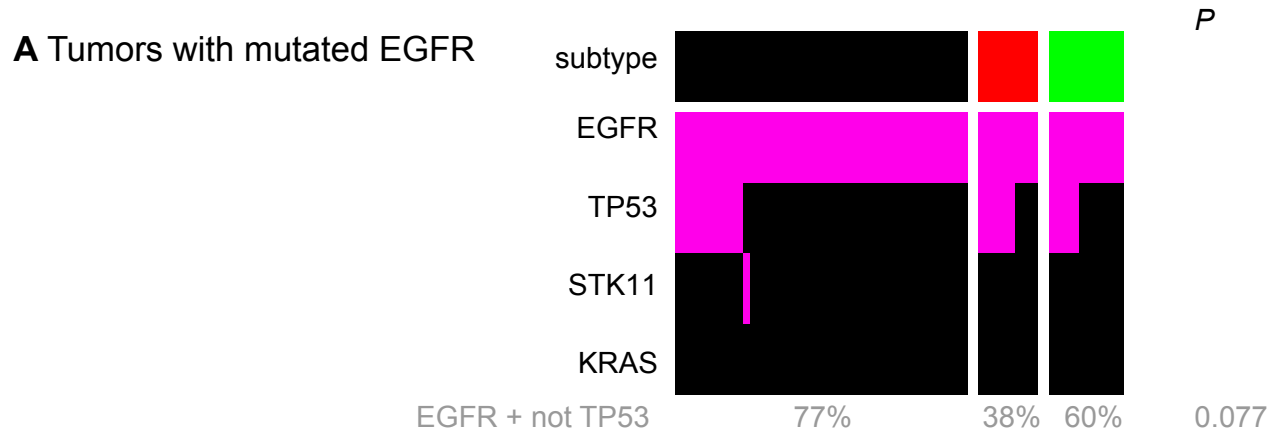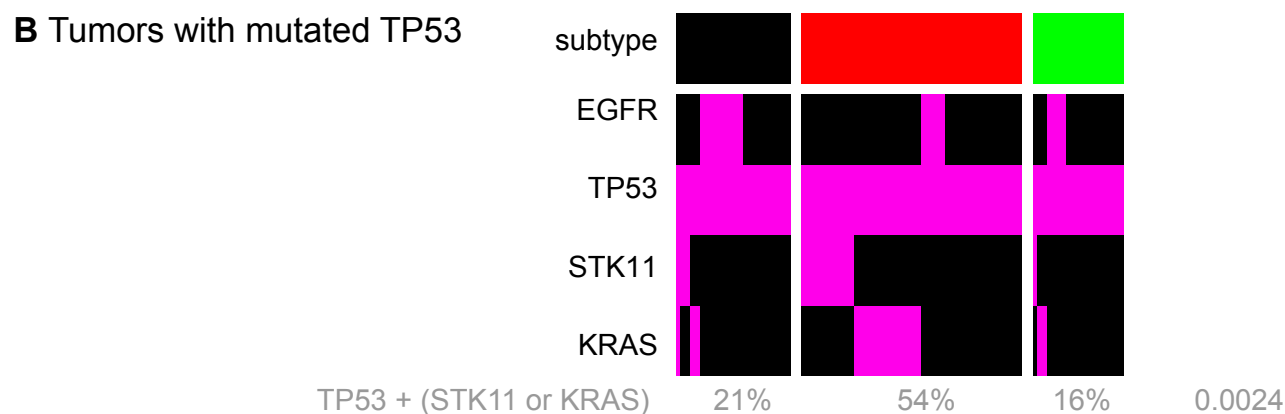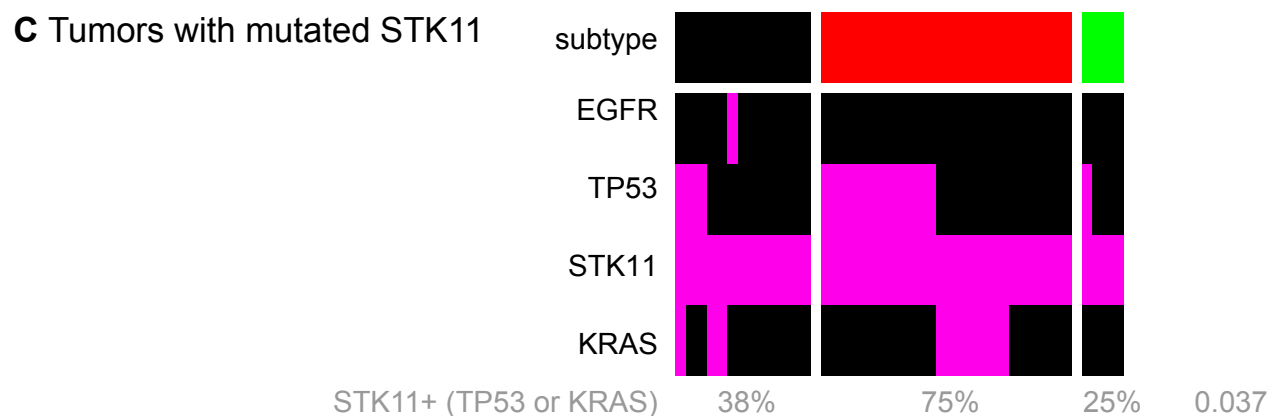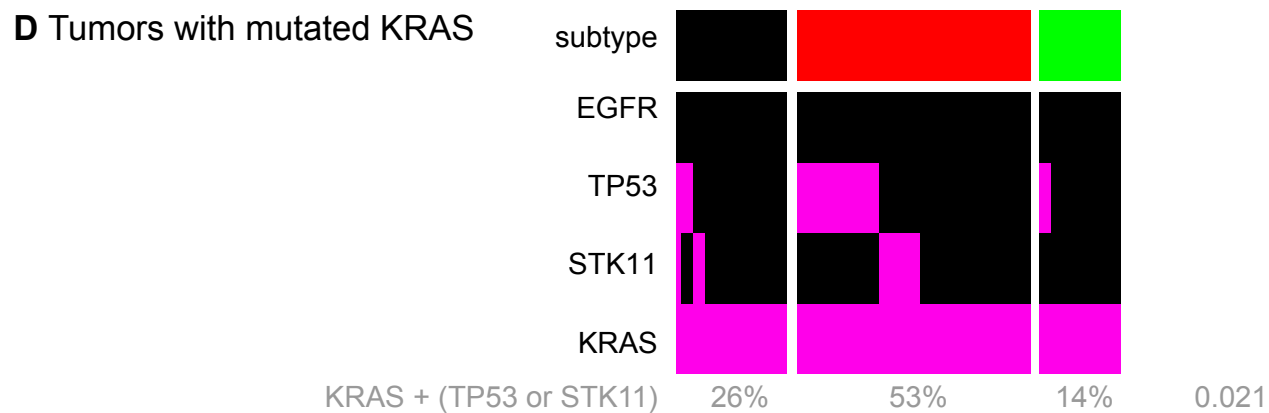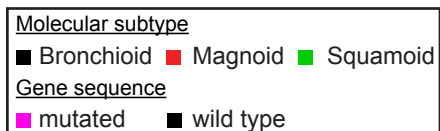

Supplement: Figure S3 — Gene sequence co-mutations. Tumors are represented as columns and genes as rows. Tumors are from all cohorts with corresponding assays (Chitate et al., Ding et al., UNC). Percentages of tumors within a subtype having a particular gene mutation combination are listed. Associations of the integrated combination with subtype were tested by Fisher's exact tests (P). (PDF) [file pone.0036530.s003.pdf]
