# Supplementary material for: Differential Pathogenesis of Lung Adenocarcinoma Subtypes Involving Sequence Mutations, Copy Number, Chromosomal Instability, and Methylation
Source: PLoS One. 2012 May 10;7(5):e36530. doi: 10.1371/journal.pone.0036530 (PMC3349715; doi:10.1371/journal.pone.0036530)
Supplement: Table S1 — Cohort data types and patient counts. Patients common to Shedden et al. and Chitale et al. cohorts (n = 88) and those common to the Zhu et al. and Shedden et al. cohorts (n = 43) were counted once in the unique adenocarcinoma and lung cancer totals. Patients common to the Tomida et al. and Takeuchi et al. cohorts (n = 26) were counted once in the unique lung cancers total. Gene sequencing refers to the number of tumors with at least one gene sequenced. (DOC) [file pone.0036530.s004.doc]

**Table S1: Cohort data types and patient counts.**

| **Cohort** | **Data type** | **Platform** | **Number of lung cancer patients** | **Number of normal lung specimens** | **Reference** |
| --- | --- | --- | --- | --- | --- |
| *Lung adenocarcinoma* |  |  |  |  |  |
| UNC | gene expression | Agilent 44K | 116 | - | GSE36471 |
|  | DNA copy number | Affymetrix 250K Sty | 56 | 20 | GSE36471 |
|  | DNA copy number | Affymetrix SNP6 | 47 | 10 | GSE36471 |
|  | methylation | Affymetrix 250K Sty (MSNP) | 33 | 10 | GSE36471 |
|  | gene sequencing | 4 genes | 109 | - | GSE36471 |
| Bhattacharjee et al. | gene expression | Affymetrix 95av2 | 128 | - | [1] |
|  | gene sequencing | 2 genes | 128 | - |  |
| Chitale et al. | gene expression | Affymetrix U133A | 90 | - | [2] |
|  | gene expression | Affymetrix U133A2 | 102 | - | [2] |
|  | DNA copy number | Agilent 44K aCGH | 192 | - | [2] |
|  | gene sequencing | 8 genes | 192 | - | [2] |
| Ding et al. | gene expression | Affymetrix U133Plus2 | 68 | - | GSE12667 |
|  | DNA copy number | Affymetrix 250K Sty | 67 | 67 | [3] |
|  | gene sequencing | 623 genes | 41 | - | [3] |
| Shedden et al. | gene expression | Affymetrix U133A | 443 | - | [4] |
| Tomida et al. | gene expression | Agilent 44K | 117 | - | GSE13213 |
|  | gene sequencing | 3 genes | 117 | - | GSE13213 |
| Zhu et al. | gene expression | Affymetrix U133A | 71 | - | GSE14814 |
| *Other lung cancer morphologies* |  |  |  |  |  |
| UNC-nonAD | gene expression (squamous cell carcinoma and large cell carcinoma) | Agilent 44K | 56,9 | - | GSE17710 |
| Takeuchi et al. | gene expression (bronchioloalveolar, large cell and squamous cell carcinoma) | Agilent 21.6K | 26, 20, 35 | - | GSE11969 |
|  |  |  |  |  |  |
|  | **Unique lung adenocarcinomas** | **Unique lung cancers** | **All samples** |  |  |
| Patients | 1,004 | 1,124 | 1,281 |  |  |
| gene expression arrays | 1,004 | 1,124 | 1,281 |  |  |
| DNA copy number arrays | 362 | 362 | 459 |  |  |
| DNA methylation (MSNP) | 33 | 33 | 43 |  |  |
| gene sequencing | 587 | 587 | 587 |  |  |
|  |  |  |  |  |  |
| Published cohorts with gene sequencing or DNA copy number arrays | 504 |  |  |  |  |

[1] http://www.broadinstitute.org/mpr/lung/

[2] http://cbio.mskcc.org/Public/lung_array_data/

[3] http://www.ncbi.nlm.nih.gov/projects/gap/cgi-bin/study.cgi?study_id=phs000144.v1.p1

[4] https://array.nci.nih.gov/caarray/project/details.action?project.experiment.publicIdentifier=jacob-00182
