# Supplementary material for: Differential Pathogenesis of Lung Adenocarcinoma Subtypes Involving Sequence Mutations, Copy Number, Chromosomal Instability, and Methylation
Source: PLoS One. 2012 May 10;7(5):e36530. doi: 10.1371/journal.pone.0036530 (PMC3349715; doi:10.1371/journal.pone.0036530)
Supplement: Table S2 — Study design. This table presents the order in which steps were followed and which datasets were used. ‘X’ indicates a cohort was used for a particular step. Separate platforms within a cohort were gene median centered separately and merged (*). (DOC) [file pone.0036530.s005.doc]

**Table S2: Study design.**

|  |  |  | Shedden et al. | Takeuchi et al. | UNC-nonAD | Bhattacharjee et al. | Chitale et al. | Ding et al. | Tomida et al. | UNC | Zhu et al. |  |
| --- | --- | --- | --- | --- | --- | --- | --- | --- | --- | --- | --- | --- |
| **Methods for generating starting data for study** | | | | | | | | | | | | |
| **A. Assay data processing** | |  |  |  |  |  |  |  |  |  |  |  |
|  | *Gene expression microarrays* |  |  |  |  |  |  |  |  |  |  |  |
| 1 | map probes to common gene set |  | X | X | X | X | X | X | X | X | X |  |
| 2 | process expression data |  |  |  |  |  |  |  |  |  |  |  |
|  | Robust Multiarray average |  | X |  |  | X | X | X |  |  | X |  |
|  | Normexp background correction,  lowess normalization |  |  | X | X |  |  |  | X | X |  |  |
| 3 | gene median center |  | X | X | X | X | X* | X | X | X | X* |  |
| 4 | *DNA copy number (CN) microarrays see Table S3* |  |  |  |  |  | X | X |  | X |  |  |
| 5 | *Methylation microarrays see Table S3* |  |  |  |  |  |  |  |  | X |  |  |
| **B. Identify subtypes and clinical characteristics** | |  |  |  |  |  |  |  |  |  |  |  |
| 1 | detect expression subtypes |  | X |  |  |  |  |  |  |  |  | Fig. S1 |
| 2 | compare subtypes to Hayes et al. subtypes and other lung morphologies |  | X | X | X | X |  |  |  |  |  | Fig. S2 |
| 3 | make subtype predictor |  | X |  |  |  |  |  |  |  |  |  |
| 4 | predict subtypes in each cohort |  | X |  |  | X | X | X | X | X | X | Fig. 1 |
| 5 | analyze clinical characteristics associated with subtype |  | X |  |  | X | X | X | X | X | X | Table 1 |
|  | | | | | | | | | | | | |
| **Study Design** | | | | | | | | | | | | |
| **Primary outcomes** | | | | | | | | | | | | |
| **C. Gene mutation associations with subtype** | |  |  |  |  |  |  |  |  |  |  |  |
| 1 | Select published tumors with gene mutations |  |  |  |  | X | X | X | X |  |  |  |
| 2 | Detect gene mutations associations |  |  |  |  | X | X | X | X |  |  | Table 2, Table S5 |
| 3 | Validate gene mutation associations |  |  |  |  |  |  |  |  | X |  | Table 2, Table S5 |
| **D. DNA copy number (CN) associations with subtype** | |  |  |  |  |  |  |  |  |  |  |  |
| 1 | Select published tumors with CN |  |  |  |  |  | X | X |  |  |  |  |
| 2 | Detect CN associations |  |  |  |  |  | X | X |  |  |  | Fig. 2 |
| 3 | Validate CN associations |  |  |  |  |  |  |  |  | X |  | Fig. 2 |
| **E. Methylation associations with subtype** | |  |  |  |  |  |  |  |  |  |  |  |
| 1 | Detect methylation associations |  |  |  |  |  |  |  |  | X |  | Fig. 4A, Table S8 |
| **Secondary outcomes** | | | | | | | | | | | | |
| **F. Integrated analysis of copy number and expression** | |  |  |  |  |  |  |  |  |  |  |  |
| 1 | Select tumors with copy number |  |  |  |  |  | X | X |  | X |  |  |
| 2 | Scale genes’ expression  divide by genes’ standard deviations by cohort |  |  |  |  |  | X | X |  | X |  |  |
| 3 | Scale platform CN  divide by platform standard deviation by cohort |  |  |  |  |  | X | X |  | X |  |  |
| 4 | Pool cohorts and analyze |  |  |  |  |  | X | X |  | X |  | Fig. 3 |
| **G. Integrated analysis of copy number and methylation** | |  |  |  |  |  |  |  |  |  |  |  |
| 1 | Select tumors with methylation and CN |  |  |  |  |  |  |  |  | X |  |  |
| 2 | Analyze CN and methylation |  |  |  |  |  |  |  |  | X |  | Fig. 4C |
| **H. Integrated analysis of mutations, CN, and expression** | |  |  |  |  |  |  |  |  |  |  |  |
| 1 | Select tumors with copy number, and mutation status for EGFR, KRAS, STK11, and TP53 |  |  |  |  |  | X | X |  | X |  |  |
| 2 | Scale gene expression and CN  Step F.2 and F.3 |  |  |  |  |  | X | X |  | X |  |  |
| 3 | Median center CIN values and scale by dividing by standard deviation by cohort |  |  |  |  |  | X | X |  | X |  |  |
| 4 | Pool cohorts and analyze |  |  |  |  |  | X | X |  | X |  | Fig. 5 |
| **I. Patient outcomes** | |  |  |  |  |  |  |  |  |  |  |  |
| 1 | Pool cohorts and analyze overall survival |  | X |  |  | X | X |  | X | X | X | Fig. 6A |
| 2 | Compare subtypes by disease-specific survival by treatment group |  |  |  |  |  |  |  |  |  | X | Fig. 6B |
| **J. Integrated analysis of EGFR mutation and gefitinib sensitivity** | | |  |  |  |  |  |  |  |  |  |  |
| 1 | Select tumors with EGFR mutation status |  |  |  |  | X | X | X | X | X |  |  |
| 2 | Scale gene expression  Step F.2 |  |  |  |  | X | X | X | X | X |  |  |
| 3 | Pool cohorts and analyze |  |  |  |  | X | X | X | X | X |  | Fig. 6C |
