# Supplementary material for: Differential Pathogenesis of Lung Adenocarcinoma Subtypes Involving Sequence Mutations, Copy Number, Chromosomal Instability, and Methylation
Source: PLoS One. 2012 May 10;7(5):e36530. doi: 10.1371/journal.pone.0036530 (PMC3349715; doi:10.1371/journal.pone.0036530)
Supplement: Table S3 — DNA copy number and methylation microarray processing. The copy number (CN) and methylation microarray processing steps for each cohort are listed. ‘X’ indicates the step was followed. Affymetrix SNP arrays were processed by CRMAv2 [46]. The Affymetrix SNP6 microarrays were subjected to an outlier probe removal method to remove nonhybridizations similar to published methods [47]. Common probe locations were calculated by taking the median of probe CN values every 2 megabases. (DOC) [file pone.0036530.s006.doc]

**Table S3: DNA copy number and methylation microarray processing.**

|  | **Copy number microarrays** | | | | **Methylation microarrays** |
| --- | --- | --- | --- | --- | --- |
|  | **Ding et al.** | **Chitale et al.** | **UNC** | **UNC** | **UNC** |
| **Platform** | Affymetrix 250K Sty | Agilent 44K aCGH | Affymetrix 250K Sty | Affymetrix SNP6 | Affymetrix 250K Sty |
| **Processing steps** |  |  |  |  |  |
| Process signal intensity | CRMAv2 | Taken as published | CRMAv2 | CRMAv2 | CRMAv2 |
| Log_2_ convert | X |  | X | X |  |
| Sample median center | X |  | X | X |  |
| Normal estimate | Normal lung specimen mean | Normal reference (as given in published data) | Normal lung specimen mean | Normal lung specimen mean |  |
| CN value:  log_2_ (tumor sample / normal estimate) | X | X (taken as published) | X | X |  |
| Sample median center |  | X |  |  |  |
| Smooth CN by Circular Binary Segmentation | X | X | X | X |  |
| Tumor autosome CN median center | X | X | X | X |  |
| Remove outlier probes |  |  |  | X |  |
| **For recurrent region identification:** |  |  |  |  |  |
| Convert to common probe locations | X | X |  |  |  |
| Standardize CN by division by standard deviation | X | X |  |  |  |
| Pool cohorts and identify recurrent CN regions by DiNAMIC | X | X |  |  |  |
| **For assigning region CN values to samples** |  |  |  |  |  |
| Standardize CN by division by standard deviation | X | X | X | X |  |
| Assign region CN value to each sample by taking median of platform’s probes in region | X | X | X | X |  |
| **For MSNP analysis** |  |  |  |  |  |
| Center samples using control site probes |  |  |  |  | X |
| Methylation site value:  (undigested – HpaII) / undigested |  |  |  |  | X |
| Assign genomewide methylation value to samples by median of methylation sites |  |  |  |  | X |
