# Supplementary material for: Differential Pathogenesis of Lung Adenocarcinoma Subtypes Involving Sequence Mutations, Copy Number, Chromosomal Instability, and Methylation
Source: PLoS One. 2012 May 10;7(5):e36530. doi: 10.1371/journal.pone.0036530 (PMC3349715; doi:10.1371/journal.pone.0036530)
Supplement: Table S4 — Validation cohort gene sequencing regions. These regions were sequenced in the UNC cohort by Polymorphic, Inc (Almeda CA) on ABI 3730XL DNA sequencers. Coordinates are from human genome assembly hg 18. Regions include some flanking intronic sequence. (DOC) [file pone.0036530.s007.doc]

**Table S4: Validation cohort gene sequencing regions.**

| **Gene** | **Exon number** | **Chromosome** | **Start** | **Stop** |
| --- | --- | --- | --- | --- |
| ***EGFR*** |  |  |  |  |
|  | 17 | 7 | 55208120 | 55208369 |
|  | 18 | 7 | 55209070 | 55209269 |
|  | 19 | 7 | 55209859 | 55210058 |
|  | 20 | 7 | 55216473 | 55216672 |
|  | 21 | 7 | 55226884 | 55227083 |
|  | 22 | 7 | 55227891 | 55228090 |
| ***KRAS*** |  |  |  |  |
|  | 2 | 12 | 25289431 | 25289630 |
| ***STK11*** |  |  |  |  |
|  | 1 | 19 | 1157913 | 1158202 |
|  | 2 | 19 | 1169358 | 1169557 |
|  | 3 | 19 | 1170268 | 1170467 |
|  | 4 | 19 | 1171339 | 1171538 |
|  | 5 | 19 | 1171549 | 1171748 |
|  | 6 | 19 | 1172176 | 1172375 |
|  | 7 | 19 | 1172877 | 1173076 |
|  | 8 | 19 | 1173978 | 1174177 |
|  | 9 | 19 | 1177450 | 1177649 |
| ***TP53*** |  |  |  |  |
|  | 5 | 17 | 7519088 | 7519287 |
|  | 6 | 17 | 7518859 | 7519058 |
|  | 7 | 17 | 7518179 | 7518378 |
|  | 8 | 17 | 7517713 | 7517912 |
