# Supplementary material for: Differential Pathogenesis of Lung Adenocarcinoma Subtypes Involving Sequence Mutations, Copy Number, Chromosomal Instability, and Methylation
Source: PLoS One. 2012 May 10;7(5):e36530. doi: 10.1371/journal.pone.0036530 (PMC3349715; doi:10.1371/journal.pone.0036530)
Supplement: Table S5 — Subtype gene sequence mutation counts. Numbers of patients with mutant or not mutant (wild type) genes are listed by subtype. These numbers correspond to the percentages in Table 2. Patient counts vary by gene because not all cohorts sequenced all tumors for all genes. (DOC) [file pone.0036530.s008.doc]

**Table S5:** Subtype gene sequence mutation counts.

|  | **Discovery cohorts**  (Bhattacharjee et al., Chitale et al., Ding et al., Tomida et al.) | | | **Validation cohort**  (UNC) | |  |
| --- | --- | --- | --- | --- | --- | --- |
|  |  | | |  | |  |
|  | # patients mutated / # patients not mutated | | | # patients mutated / # patients not mutated | | |
|  |  | | |  | |  |
| **Gene** | **Bronchioid** | **Magnoid** | **Squamoid** | **Bronchioid** | **Magnoid** | **Squamoid** |
|  |  | | |  |  |  |
| *EGFR* | 69/116 | 16/158 | 22/97 | 9/29 | 2/26 | 1/16 |
| *KRAS* | 30/155 | 52/122 | 21/98 | 2/42 | 14/24 | 4/23 |
| *STK11* | 13/88 | 17/62 | 4/49 | 1/36 | 8/20 | 0/17 |
| *TP53* | 31/111 | 49/73 | 28/57 | 4/38 | 22/16 | 4/23 |
| *BRAF* | 5/96 | 0/79 | 1/52 | - | - | - |
| *LRP1B* | 0/16 | 3/15 | 2/5 | - | - | - |
| *PTEN* | 3/98 | 2/77 | 4/49 | - | - | - |
