# Supplementary material for: Differential Pathogenesis of Lung Adenocarcinoma Subtypes Involving Sequence Mutations, Copy Number, Chromosomal Instability, and Methylation
Source: PLoS One. 2012 May 10;7(5):e36530. doi: 10.1371/journal.pone.0036530 (PMC3349715; doi:10.1371/journal.pone.0036530)
Supplement: Table S6 — Subtype genomewide mutation rates. Non-synonymous genome wide rates were calculated for each tumor by diving the number of non-synonymous mutations by the number of nucleotides sequenced. Mutation rates were significantly different (Kruskal-Wallis test two-sided P<0.01). Confidence intervals were calculated by 1,000 bootstrap replicates. (DOCX) [file pone.0036530.s009.docx]

**Table S6. Subtype genomewide mutation rates.**

|  | Genomewide mutation rates (mutations per megabase) | | | | | |
| --- | --- | --- | --- | --- | --- | --- |
|  | Mean |  | 95% Confidence Internal | | | |
| Bronchioid | 0.509 | ( | 0.182 | - | 0.926 | ) |
| Magnoid | 1.52 | ( | 0.952 | - | 2.31 | ) |
| Squamoid | 1.45 | ( | 0.249 | - | 2.86 | ) |
