# Supplementary material for: Differential Pathogenesis of Lung Adenocarcinoma Subtypes Involving Sequence Mutations, Copy Number, Chromosomal Instability, and Methylation
Source: PLoS One. 2012 May 10;7(5):e36530. doi: 10.1371/journal.pone.0036530 (PMC3349715; doi:10.1371/journal.pone.0036530)
Supplement: Table S7 — Regions of recurrent DNA copy number amplifications and deletions. Regions of recurrent DNA copy number amplifications and deletions were calculated by DiNAMIC 32] (P<0.001). CN amplifications and deletions are indicated by 1 and –1, respectively. For each region, marker refers to the point of the most extreme CN, and left and right refer to the region's boundaries. Positions are hg 18 genomic coordinates. (DOC) [file pone.0036530.s010.doc]

**Table S7: Regions of recurrent DNA copy number amplifications and deletions.**

| **Region** | **Amplification/Deletion** | **Chromosome** | **Marker** | **Left position** | **Right position** |
| --- | --- | --- | --- | --- | --- |
| 5p15 | 1 | 5 | 2244437 | 2244437 | 8036035 |
| 1q21-23 | 1 | 1 | 149214459 | 145217965 | 163222759 |
| 8q23-24 | 1 | 8 | 129280590 | 117657603 | 135351868 |
| 7p22-12 | 1 | 7 | 25819282 | 2580308 | 48128151 |
| 14q13-21 | 1 | 14 | 36308939 | 36308939 | 38540928 |
| 12q14-21 | 1 | 12 | 66917589 | 65150190 | 70905077 |
| 20q13 | 1 | 20 | 61848793 | 52526436 | 61848793 |
| 8p23-12 | -1 | 8 | 23402634 | 1587779 | 33389345 |
| 7q11-22 | 1 | 7 | 86431171 | 62290707 | 100546587 |
| 15q11-21 | -1 | 15 | 18872997 | 18872997 | 44055665 |
| 13q12-21 | -1 | 13 | 18720011 | 18720011 | 56776786 |
| 18q12-23 | -1 | 18 | 48134907 | 32233439 | 74030896 |
| 17q23-25 | 1 | 17 | 76698689 | 55144988 | 76698689 |
| 9p24-21 | -1 | 9 | 23188776 | 1278937 | 27255650 |
| 6q14-23 | -1 | 6 | 100870643 | 78932689 | 133092983 |
| 3p26-12 | -1 | 3 | 56767319 | 999266 | 86866056 |
| 9q12-34 | -1 | 9 | 66256186 | 66256186 | 131378950 |
| 16p13-11 | 1 | 16 | 31277953 | 1710513 | 31277953 |
| 19p13 | -1 | 19 | 6177006 | 2571168 | 15858564 |
| 19q12-13 | 1 | 19 | 36232246 | 34188203 | 40153045 |
| 6p25-11 | 1 | 6 | 45061736 | 1162500 | 58716531 |
| 11q13 | 1 | 11 | 69353729 | 65380601 | 69353729 |
| 2p25-22 | 1 | 2 | 28212267 | 2108078 | 37789864 |
| 20p12-11 | 1 | 20 | 25533911 | 16280675 | 25533911 |
| 5q33-35 | 1 | 5 | 171947678 | 157925257 | 178143455 |
| 8p12-11 | 1 | 8 | 41277161 | 37219919 | 41277161 |
| 2q22-35 | 1 | 2 | 171332216 | 141968319 | 220132731 |
| 1p35-32 | 1 | 1 | 43091859 | 33551051 | 53378570 |
| 17p13-11 | -1 | 17 | 6743364 | 1012577 | 17066766 |
| 5q11 | 1 | 5 | 50319301 | 50319301 | 53963835 |
| 22q13 | -1 | 22 | 47036414 | 38810812 | 48313502 |
| 10q22-q26 | -1 | 10 | 88570230 | 78950016 | 132646770 |
| 4q33-35 | -1 | 4 | 185371215 | 171292085 | 189359445 |
| 3q26 | 1 | 3 | 170651000 | 166926709 | 182965497 |
| 5q13-31 | -1 | 5 | 70896334 | 67777495 | 137965439 |
| 12q21 | -1 | 12 | 81078153 | 73346321 | 84659823 |
| 14q23-32 | -1 | 14 | 98422714 | 58505357 | 106210547 |
| 1p31-22 | -1 | 1 | 91031160 | 74901095 | 93002576 |
| 8q11 | -1 | 8 | 47689419 | 47689419 | 53425138 |
