# Supplementary material for: Differential Pathogenesis of Lung Adenocarcinoma Subtypes Involving Sequence Mutations, Copy Number, Chromosomal Instability, and Methylation
Source: PLoS One. 2012 May 10;7(5):e36530. doi: 10.1371/journal.pone.0036530 (PMC3349715; doi:10.1371/journal.pone.0036530)
Supplement: Table S8 — Differentially methylated site totals. Cells contain the number of sites with significantly greater methylation in the row class compared to the column class. Methylation was compared between classes by Wilcoxon rank-sum tests that evaluated the null hypothesis of not greater methylation in the row class (Benjamini-Hochberg adjusted P<0.05). For example, 11,720 sites had significantly greater methylation in Magnoid tumors compared to normal lung. Squamoid and Bronchioid hypermethylated sites compared to normal lung were almost completely contained in the Magnoid versus normal sites (83% and 88%, respectively). (DOC) [file pone.0036530.s011.doc]

**Table S8: Differentially methylated site totals**.

|  |  | **Specimen class** | |  |  |
| --- | --- | --- | --- | --- | --- |
|  |  | Bronchioid | Squamoid | Magnoid | Normal lung |
| **Specimen class** | Bronchioid | - | 0 | 0 | 1,272 |
|  | Squamoid | 0 | - | 0 | 2,267 |
|  | Magnoid | 74 | 50 | - | 11,720 |
|  | Normal lung | 1,886 | 886 | 920 | - |
